# Prevalence of vitamin D deficiency in the pregnant women: an observational study in Shanghai, China

**DOI:** 10.1186/s13690-020-00414-1

**Published:** 2020-06-04

**Authors:** Huijuan Li, Jing Ma, Runzhi Huang, Yuhua Wen, Guanghui Liu, Miao Xuan, Ling Yang, Jun Yang, Lige Song

**Affiliations:** 1 Department of Endocrinology, Tongji Hospital, Tongji University School of Medicine, Shanghai, 200065 China; 2 Division of Spine, Department of Orthopedics, Tongji Hospital affiliated to Tongji University School of Medicine, Shanghai, 200065 China; 3 Key Laboratory of Spine and Spinal Cord Injury Repair and Regeneration (Tongji University) Ministry of Education, Shanghai, 200065 China; 4 Shanghai Changning District Maternal and Child Health Hospital, Shanghai, 200065 China; 5 Tongji Hospital, Tongji University School of Medicine, Shanghai, 200092 China

**Keywords:** Vitamin D deficiency, Prevalence, Pregnancy, Observational study

## Abstract

**Background:**

Maternal vitamin D deficiency has been a worldwide concern in recent years. However the epidemiological data of vitamin D deficiency among large group of Chinese pregnant women is limited. This study is to evaluate the prevalence of vitamin D deficiency among pregnant women in Shanghai, China and to analyze the association of vitamin D status with some pregnancy outcomes (gestational diabetes and low birth weight).

**Methods:**

A total of 34,417 pregnant women in Shanghai were included in this study from January 2014 to December 2017, and the serum 25-hydroxyvitamin D [25(OH)D] concentrations were measured at 16th week of gestation by electrochemiluminescence assay. Seventy five grams of glucose was used to conduct oral glucose tolerance test during 24-28th week of gestational in all enrolled persons and the birth weight of newborns was recorded.

**Results:**

The median serum 25(OH) D concentration in the pregnant women during 4 years was 42.87 nmol/L (32.88–51.90 nmol/L). 9.9% of the population were severe vitamin D deficient [25(OH)D < 25 nmol/L], 60.1% were deficient [25 nmol/L ≤ 25(OH)D < 50 nmol/L], 28.4% were insufficient [50 nmol/L ≤ 25(OH)D < 75 nmol/L] and only 1.6% of the enrolled population reached the level of adequate [25(OH)D ≥ 75 nmol/L]. Serum 25(OH) D concentrations showed significant difference among seasons with the highest level in winter and the lowest level in summer. Women with advanced maternal age were more likely to have better vitamin D status compared with younger women. The 25(OH) D levels were significantly different among 2014–2017. The year of 2017 had the highest 25(OH) D level with the median serum concentration reaching 47.80 nmol/L (41.00–55.00 nmol/L), while the lowest appeared in 2016 which has median 25(OH) D concentration at 38.87 nmol/L (28.76–49.97 nmol/L). No relations were found between the 25(OH) D status and the rate of gestational diabetes or low birth weight of newborns.

**Conclusion:**

Pregnant women in Shanghai were generally deficient in vitamin D status and the level of vitamin D was related to season and age. No evidence showed vitamin D deficiency in pregnant women contributes to the rate of gestational diabetes or low birth weight of newborns in this study. These results suggest that most of the pregnant women may need vitamin D supplementation to achieve adequate vitamin D level.

## Background

Vitamin D has been recognized as one of the most important biological regulators of calcium homeostasis and it has great influence on bone and mineral metabolism in mammals. However, based on the evidence that the vitamin D receptor expressed in multiple cells and tissues and the wide distribution of 1-alpha-hydroxylase, a key enzyme for 1,25-dyhydroxyl-vitamin D production, more and more studies have shown that the role of vitamin D is not limited to bone health [1]. Vitamin D can regulate immune function [2], hematopoietic system [3], cancer development and progression [4], glucose homeostasis through insulin production and insulin resistance [5] and cognitive function [6]. Thus vitamin D deficiency may be related to many diseases, such as asthma [7], diabetes mellitus [8], cardiovascular diseases [9], cancers [10, 11].

Epidemiological data in recent years have shown that vitamin D deficiency is widespread in the population, including pregnant women. A retrospective cohort study conducted in USA with 235 cases reported 30% of the pregnant women had adequate vitamin D level with serum 25(OH) D concentration more than 75 nmol/L [12]. Result from a national cross-sectional survey in Belgium revealed that 25.9% of women had 25(OH) D more than 75 nmol/L [13]. And a cross-sectional observation study from China found that in 5823 cases only 0.9% of pregnant women had adequate vitamin D status with 25(OH) D more than 80 nmol/l during the second trimester of pregnancy in 2011–2012 [14]. Thus, vitamin D deficiency in pregnancy has been a worldwide problem. Maternal vitamin D deficiency may be associated with a variety of adverse pregnancy outcomes, including pre-eclampsia [15, 16], preterm birth [17], gestational diabetes mellitus [15, 18] and bacterial vaginitis during pregnancy [15, 19]. The vitamin D level in fetus is exclusively dependent on the mother so maternal 25(OH) D level can potentially impact fetal development [20–22]. The offspring of pregnant women who are vitamin D deficient may have high risks of small for gestational age [15, 23], low birth weight [15, 23] and respiratory infections [19].

Recently more and more side effects of vitamin D deficiency have been recognized, and more attention has been paid on the vitamin D deficient pregnant women. But whether vitamin D nutritional status in Chinese pregnant women has improved during recent years is not clear. This observational study was designed to evaluate the prevalence of vitamin D deficiency at 16th week of gestation among a large group of pregnant women in Shanghai and the associations of vitamin D deficiency with some pregnancy outcomes (gestational diabetes and low birth weight) were analyzed, and hope to provide epidemiological evidence of vitamin D deficiency in Chinese pregnant women.

## Methods

### Study population

Participants who joined the regular pregnancy check-up in Shanghai Changning District Maternal and Child Health Hospital from January 2014 to December 2017 were recruited. This hospital located in Shanghai, the eastern China with the latitude at 31.1°N. This hospital accepted pregnant women for prenatal care around all the districts of Shanghai. All the pregnant women who came to this hospital for prenatal examination at 16th week gestation were invited to test the vitamin D status. And we collected the test results retrospectively. Gestational age was calculated by the last menstrual date. The inclusion criteria of this study includes: (1) Han nationality; (2) lived in Shanghai for more than 1 year. The exclusion criteria includes: (1) chronic metabolic diseases affecting the metabolism of vitamin D, such as hyperthyroidism, hyperparathyroidism, chronic kidney disease and liver failure, etc.; (2) mental diseases; (3) either of the pregnant woman or her spouse had been diagnosed congenital deformities or genetic metabolic diseases; (4) either of the pregnant woman or her spouse had been addicted to smoking, alcohol or drug before or during the pregnancy. The Ethics Committee of Tongji Hospital, Tongji University School of Medicine declared that no formal ethics approval was required in this particular case. This study is a retrospective and observational study, and patient consent is not signed by each patient. All examination items were derived from routine prenatal care.

### Categorization of vitamin D

25(OH) D is the most abundant circulating form of vitamin D in the human body and serum 25(OH) D concentration has be a reliable indicator to assess the status of vitamin D. According to the concentration of serum 25(OH) D, the vitamin D status can be divided into four levels: severe deficiency (< 25 nmol/L), deficiency (25-50 nmol/L), insufficiency (50-75 nmol/L) and adequate (≥75 nmol/L). And 50 nmol/L is considered as a threshold of deficiency and non-deficiency.

### Definition of seasons and age groups

The seasons of sampling were defined as spring (March, April, May), summer (June, July, August), autumn (September, October, November) and winter (December, January, February). All participants were categorized into four age groups: 18–24 years old group, 25–29 years old group, 30–35 years old group, and > 35 years old group.

### Pregnancy outcomes

Diagnosis of gestational diabetes followed the guideline demonstrated by American Diabetes Association. The glucose tolerance test was performed at 24-28th week gestation, and 75 g of glucose was given orally under fasting condition. Participant was diagnosed gestational diabetes if plasma glucose concentrations met one of the following values: ≥5.1 mmol/l on fasting, ≥10 mmol/l at 1 h and ≥ 8.5 mmol/l at 2 h. Low birth weight of newborn was defined as birth weight lower than 2500 g.

### Estimating the concentration of 25(OH)D

Blood samples were collected from all participants in fasting condition at 16th week gestation. And the samples were stored at − 80 °C until the serum 25(OH) D concentrations were measured by electrochemiluminescence assay (Roche Diagnostics, Cat. No. 05894913), and the period between blood collection and test was within 3 days. The assay has a sensitivity of 4 ng/ml, and intra and inter-assay coefficient of variation of as 6.6 and 5.4%.

### Statistical analysis

The statistical analysis was performed using Statistical Package for the Social Science (SPSS), version 20.0. Mean ± SD was used to describe continuous variables. Median, interquartile range and percentiles (p25-p75) were used to describe 25(OH) D concentrations because the distribution did not satisfy the criteria of normal distribution and non-parametric Kruskall-Wallis test was performed. Categorical variables were expressed as percentages and the proportions were compared using Chi-square test. Multiple logistic regression analysis was applied to access the relationship between relevant factors (season, age, year) and vitamin D status, and whether vitamin D status had association with the two pregnancy outcomes. *P* < 0.05 was regarded to be statistically significant for all performed tests.

## Results

Thirty-four thousand four hundred seventeen pregnant women were included in this observational study. The mean age of the participations was 30.57 ± 3.52 years (range:19-46 years). The median concentration of 25(OH) D was 42.87 nmol/L (32.88–51.90 nmol/L). 9.9% of the population were severe deficient (< 25 nmol/L), 60.1% were deficient (25-50 nmol/L), 28.4% were insufficient (50-75 nmol/L) and only 1.6% of the subjects were adequate (≥75 nmol/L) (Table 1).

**Table 1:** Levels of serum 25(OH) D concentrations among different seasons, age groups and years

Group	N	Median (Interquartile Range) (nmol/L)	Percentile (p25-p75) (nmol/L)	*P*-value	Severe deficiency (< 25 nmol/L) (%)	Deficiency (25-50 nmol/L) (%)	Insufficiency (50-75 nmol/L) (%)	Adequate (≥75 nmol/L) (%)	*P*-value
**Season**				< 0.001					< 0.001
Spring	8299	42.00 (19.73)	(31.07–50.80)		11.9	60.6	26.4	1.1	
Summer	10,111	41.76 (18.20)	(32.60–50.80)		10.7	62.1	25.4	1.8	
Autumn	9188	43.13 (17.80)	(34.30–52.10)		7.8	61.5	29.4	1.2	
Winter	6819	45.10 (21.67)	(33.28–54.96)		9.2	54.4	33.8	2.6	
**Categorical age**				< 0.001					< 0.001
18–24	782	39.42 (17.82)	(30.99–48.81)		11.9	66.6	20.2	1.3	
25–29	14,070	42.00 (19.22)	(31.84–51.06)		10.9	61.2	26.6	1.4	
30–35	16,283	43.46 (18.77)	(33.75–52.53)		9.1	59.4	29.7	1.8	
> 35	3282	44.19 (19.28)	(34.00–53.28)		9.2	57.2	31.7	2.0	
**Year**				< 0.001					< 0.001
2014	6459	41.45 (17.96)	(32.97–50.93)		5.6	66.9	26.6	1.0	
2015	8639	39.30 (20.49)	(29.67–50.17)		13.8	60.8	23.7	1.7	
2016	9740	38.87 (21.21)	(28.76–49.97)		17.1	58.0	23.0	2.0	
2017	9579	47.80 (14.00)	(41.00–55.00)		2.1	56.9	39.3	1.7	
**Total**	34,417	42.87 (19.02)	(32.88–51.90)		9.9	60.1	28.4	1.6	

Serum 25(OH) D concentration between seasons was significant different analyzed by Kruskal-Wallis test (*P* < 0.001). And different proportion of categorical 25(OH) D levels was found by Chi-square test (P < 0.001). Compared to the winter season, serum 25(OH) D level was lower in summer. The median concentration of 25(OH) D in summer was 41.76 nmol/L (32.60–50.80 nmol/L), which was the lowest, with 10.7% of vitamin D severe deficiency and 62.1% of deficiency in the pregnant women respectively and only 1.8% of women had adequate vitamin D. The highest 25(OH) D concentration occurred in winter and the median concentration was 45.10 nmol/L (33.28–54.96 nmol/L), with 9.2% of vitamin D severe deficiency and 54.4% of deficiency (Table 1).

Kruskal-Wallis test (*P* < 0.001) and Chi-square test (P < 0.001) both revealed the significant difference of vitamin D levels between different age groups. The > 35 years old group had the highest vitamin D level with 44.19 nmol/L (34.00–53.28 nmol/L) followed by 30–35 years old group and 25–29 years old group, and the 18–24 years old group had the lowest vitamin D level. The percentage of pregnant women with severe deficiency and deficiency in > 35 years old group was lower than the other groups (Table 1).

Vitamin D levels were significantly different between years. The serum 25(OH) D had the highest level in the year of 2017 and the lowest in 2016. The median concentration was 47.80 nmol/L (41.00–55.00 nmol/L) in 2017 and 38.87 nmol/L (28.76–49.97 nmol/L) in the year of 2016. Percentages of sever deficiency and deficiency were 2.1 and 56.9% respectively in 2017, and both of them were lower than the other years (Table 1).

The multiple logistic regression analysis demonstrated that season, categorical age and year were correlated with 25(OH) D status, all *P* < 0.001 except for the season of summer. Compared with spring season, autumn and winter had lower risk of vitamin D deficiency, and the odds ratios were 0.841 and 0.607 respectively. Elder age could significantly decrease the risk of vitamin D deficiency and the odds ratios of 25–29, 30–35, > 35 years old groups were 0.673, 0.577, and 0.550 respectively compared to the 18–24 years old group. Compared with the year of 2014, the odds ratios of the year of 2015, 2016, and 2017 were 1.186, 1.224, and 0.554 respectively (Table 2).

**Table 2:** Multiple logistic regression analysis of factors for vitamin D deficiency

Predictors	*P*-value	Odds ratio	95% confidence interval
Season			
Spring	–	1.000	Reference
Summer	0.451	1.026	0.960–1.096
Autumn	< 0.001	0.841	0.786–0.899
Winter	< 0.001	0.607	0.566–0.652
Categorical age			
18–24	–	1.000	Reference
25–29	< 0.001	0.673	0.564–0.803
30–35	< 0.001	0.577	0.484–0.688
> 35	< 0.001	0.550	0.456–0.664
Year			
2014	–	1.000	Reference
2015	< 0.001	1.186	1.101–1.277
2016	< 0.001	1.224	1.139–1.316
2017	< 0.001	0.554	0.517–0.594

As to the pregnancy outcomes, both of gestational diabetes and low birth weight had no significant differences between vitamin D deficient and non-deficient groups according to the results of Chi-square test (Table 3). And no significant association was found between the 25(OH) D status and gestational diabetes or low birth weight by logistic regression analysis after adjusted for maternal age (Table 4).

**Table 3:** Rate of gestational diabetes and low birth weight in vitamin D deficient and non-deficient groups

Pregnancy outcomes (n,%)	Overall subjects (*n* = 34,417)	25(OH) D < 50 nmol/L (*n* = 24,084)	25(OH) D ≥ 50 nmol/L (*n* = 10,333)	*P*-value
Gestational diabetes	4227 (12.3%)	2943 (12.2%)	1284 (12.4%)	0.593
Low birth weight	559 (1.6%)	387 (1.6%)	172 (1.7%)	0.706

**Table 4:** Multiple logistic regression analysis of relationship between vitamin D level and pregnancy outcomes

Pregnancy outcomes	*P*-value	Odds ratio	95% confidence interval
Gestational diabetes	0.530	1.023	0.953–1.098
Low birth weight	0.796	0.976	0.814–1.171

## Discussion

This study was conducted in a large group of samples with 34,417 Han participants to evaluate the prevalence of vitamin D deficiency in pregnant women in Shanghai, China. Our results revealed that most of the pregnant women (98.4%) did not achieve adequate vitamin D status and 70% of them were severe deficient or deficient.

Latitude might be one of the important factors which contributes to the variation of 25(OH) D status between cities [24]. A previous study involving 1695 patients carried out in Shanghai from July 2008 to June 2009 showed that the average concentration was 17.57 ng/mL (43.93 nmol/L, conversion of 25(OH)D: 1 ng/mL = 2.5 nmol/L) [25], which was quite consistent to our result. A study with 1924 patients enrolled from Nanjing (latitude 32.0°N), a city almost at the same latitude as Shanghai, indicated that the median serum 25(OH) D was 43.4 nmol/L in the second pregnancy trimester [26]. Result of a study conducted in Guangzhou, a city in southern China (latitude 23.1°N) revealed that the mean serum 25(OH) D during pregnancy was 27.03 ng/mL (67.58 nmol/) [27] which is higher than Shanghai in our study, while another study from Beijing, a city in northern China (latitude 39.9°N) found that the mean concentration of pregnant women was only 28.64 nmol/L [28], which is lower than our result. The above data shows vitamin D deficiency in pregnant women in China is very popular, same as other countries [29–31].

Season is another factor which contribute to the level of serum 25(OH) D, usually with the highest level in summer and the lowest in winter [14, 27, 32, 33]. This can be explained by different ultraviolet radiation intensity between seasons. 25(OH) D in humans can be converted from 7-dehydrocholesterol in the skin by the photochemical action of ultraviolet B (UVB, 290-315 nm wavelength) radiation, which is the main source of vitamin D in humans in general [34] and is the best marker of vitamin D status. In summer, the ultraviolet intensity is higher, thus 25(OH) D is produced more during this period. However, the seasonal variation was not always observed the same. For instance, no statistically significant difference was found among seasons in a study from USA [12]. In this study, we found that the highest 25(OH) D level appeared in winter which was contrary to some previous studies. This could be explained by other factors affecting ultraviolet radiation intensity such as cloud cover. In Shanghai, rainy day was quite frequent in June and July, called *plum rain season*. And in China, light skin was regarded as more beautiful in women so sun blockers had been extensively used in summer such as umbrella, hat and sunscreen. In addition to vitamin D converted from the skin, part of the amount of vitamin D can be derived from diet and dietary supplements as well [35]. More persons choose to increase vitamin D intake in winter to compensate for the reduced ultraviolet exposure. The reasons above may contribute to the lower 25(OH) D level in summer and narrowed the gap between median 25(OH) D concentrations in different seasons.

We also found that the participants with the elderly pregnant age had relatively higher proportion of sufficient 25(OH) D status. Pregnant women in > 35 years old group had the highest serum 25(OH) D concentration. This suggested that elderly pregnant women probably paid more attention to pregnancy care. They might expose themselves more to sunlight and more of them have vitamin D supplement from food or other multivitamins comparing to the younger pregnant women who might care more about their skin color change under sunshine. The result also revealed significant difference between years probably owning to different atmospheric contamination and overcast affecting ultraviolet intensity in these 4 years. Different exposure level to sunlight and vitamin intake might have an important effect as well. The year of 2017 had the highest level with the median serum concentration reaching 47.80 nmol/L (41.00–55.00 nmol/L) which might be attributed to the increased concern about vitamin D deficiency in recent years. However, even more attention had been paid on the vitamin D deficient in the pregnant women, most of them were still in the status of severe deficient or deficient.

Some study revealed that maternal 25(OH) D status was related with gestation diabetes and low birth weight [15, 18, 23, 27]. However, in our study, no associations were found between vitamin D status with gestation diabetes and low birth weight.

The Institute of Medicine (IOM, USA) suggests that serum 25(OH) D level superior to 20 ng/mL (50 nmol/L) can be adequate [36]. However the Endocrine Society (USA) considers the threshold level should be 30 ng/mL (75 nmol/L) [20]. And the Endocrine Society has suggested that pregnant women require at least 600 IU vitamin D per day and at least 1500–2000 IU vitamin D per day is necessary to maintain a concentration of 25(OH) D higher than 30 ng/mL (75 nmol/L) [20]. Gestational vitamin D intake may have a protective role in offspring health such as reduce the risk of multiple sclerosis [37] and other autoimmune diseases. According to the result from a cohort study of 1669 mother–child pairs in Finland, higher maternal intake of vitamin D during pregnancy could reduce the risk of allergic outcomes in the children by 5 years old [38]. Meanwhile, evidence from a small perspective study suggested that exposure to optimal 25(OH) D (> 75 nmol/L) in pregnancy could be an increased risk of atopic disorders such as eczema and asthma [39]. More research is needed to determine a reasonable vitamin D intake guideline which can promote the health of mother and child and avoid adverse effects caused by excessive vitamin D intake in the meantime. To date, there is no guideline about vitamin D supplement during gestation period in China and the clinical criterion for vitamin D deficiency in pregnant women is still uncertain and debatable.

The main limitation of this study is the lack of demographic and characteristic details of study population, including sunlight exposure status, vitamin D supplement from food and multivitamins and body mass index. Another limitaion is the 25(OH) D level in this study is detected by the Roche assay, thus the detected 25(OH) D level in the pregnant women may be lower than their real level because of the increased DBP level in the pregnant women [40]. And also we have to mentione here that an external QC for detecting 25(OH) D level with Roche assay is not available when we conducted this study, thus the differences of 25(OH) D level in this study over time may be due to drifts in the calibration or changes in the generation of Roche assays. And more information about ultraviolet intensity in 2014–2017 is needed as well. However, large sample size with 34,417 cases in this study can warn us the popularity of vitamin D deficiency in Chinese pregnant women and further research may be planned based on the results in this study.

## Conclusions

In conclusion, we found that pregnant women in Shanghai were generally deficient in vitamin D status. And temporarily there was no evidence that vitamin D deficiency had association with gestational diabetes and low birth weight. The results suggest that most of the pregnant women may need enough vitamin D supplements to achieve adequate vitamin D status and to at least partly avoid the complications related to vitamin D deficiency in the pregnant women.

## Data Availability

The datasets used and analysed during the current study are available from the corresponding author on reasonable request.
